# Soft pneumatic elbow exoskeleton reduces the muscle activity, metabolic cost and fatigue during holding and carrying of loads

**DOI:** 10.1038/s41598-021-91702-5

**Published:** 2021-06-15

**Authors:** John Nassour, Guoping Zhao, Martin Grimmer

**Affiliations:** 1 Department of Electrical and Computer Engineering, Technical University of Munich, 80333 Munich, Germany; 2 Institute of Sport Science, Technical University Darmstadt, 64289 Darmstadt, Germany

**Keywords:** Biomedical engineering, Engineering, Disease prevention, Quality of life

## Abstract

To minimize fatigue, sustain workloads, and reduce the risk of injuries, the exoskeleton Carry was developed. Carry combines a soft human–machine interface and soft pneumatic actuation to assist the elbow in load holding and carrying. We hypothesize that the assistance of Carry would decrease, muscle activity, net metabolic rate, and fatigue. With Carry providing 7.2 Nm of assistance, we found reductions of up to 50% for the muscle activity, up to 61% for the net metabolic rate, and up to 99% for fatigue in a group study of 12 individuals. Analyses of operation dynamics and autonomous use demonstrate the applicability of Carry to a variety of use cases, presumably with increased benefits for increased assistance torque. The significant benefits of Carry indicate this device could prevent systemic, aerobic, and/or possibly local muscle fatigue that may increase the risk of joint degeneration and pain due to lifting, holding, or carrying.

## Introduction

Human performance of holding and carrying loads is limited due to maximum muscle force and accumulating fatigue, which is defined as the exercise-induced reduction of the capacity to generate maximal muscle force or power output^1,2,66^. Fatigue occurs due to a variety of cellular mechanisms, such as limited energetic supply, limited transmission of the muscle action potential, or limited reuptake and release of calcium ions^3^. For greater loads, muscle fatigue occurs earlier, and thus the possible duration of holding or carrying a load will be reduced. While 15 kg can be held by the arms in front of the body for about 7 min, 40 kg can be supported for about 1 min^4^. During load carrying, holding duration is further reduced (25%^4^). Increasing loads and workspace reach levels will increase the muscle activity and metabolic cost^4–6^.

Fatigue and joint loads near physiological limits may be less harmful if sufficient breaks and regeneration times are provided. However, without this, such daily strain can cause degenerative processes that are often accompanied by pain. Occupational hazards for increased prevalence or incidence of back pain are heavy work, frequent manual load handling, occasional very stressful load handling, load handling near one’s strength capacity, reaching with extended arms, and pushing and pulling of objects^7,8^. Pain due to degeneration can also be found at the joints of the upper and lower extremities^9^. Pain of the neck and upper extremity was found to be as common as back pain (42%^10^). Occupational groups with a high incidence of back pain are nursing aides, orderlies, craft workers, machine operators, agricultural workers, and construction workers^7,10^.

To this end, the National Institute for Occupational Safety and Health (NIOSH) recommends energy expenditure limits for workers engaged in repetitive manual lifting to prevent systemic, aerobic, and possibly local muscle fatigue that may increase the risk of lifting-related pain^11^. Limits of 2.2 kcal/min and 3.1 kcal/min for lifting above and below 0.75 m, respectively, were introduced for activities lasting 2–8 h. Further, maximum load limits are recommended for a variety of lifting tasks^11^.

Additionally, solutions are required to either reduce loads or avoid muscle fatigue due to excessive loads. Exoskeletons are new technical aids that target the reduction of muscle fatigue at the trunk and the lower and upper limbs. Objectives for the trunk include spine stabilization and back support for forward bending^12,13^. Objectives for the lower limb include effort reduction for sit to stand transitions, standing, walking, climbing stairs, lifting of heavy objects, or load carrying^14–19^. Additionally, exoskeletons have been developed to enable human-like joint behavior for a single impaired joint^20^ and to enable upright locomotion for gait rehabilitation^21^. Exoskeletons for users with severe locomotion limitations include rigid, user-stabilizing structures^21^. Individuals with locomotion capabilities can instead use minimalistic and lightweight designs^22,23^, as additional weight in general, and especially increased weight at the limbs (inertia), will increase user effort^24^. Soft structures, as used in the exosuit concept^18,22,25^, seem promising to assist distal lower limb joints in daily tasks^26^. To minimize inertia-related increases of effort, exosuit actuators (motors) are placed near the body’s center of mass and Bowden cables are placed in parallel to the human muscles to transfer the assisting torques to the joints. By using a textile human–machine interface, exosuits avoid rigid mechanical linkages (in parallel with bones) and mechanical joints (in parallel with the biological joint), which can be misaligned, cause resistance, and thus increase user effort^27^.

Similar concepts from lower limb systems are being integrated into upper limb systems^28^, where objectives include lifting, lowering, holding, or carrying of objects as well as rehabilitation^28,29^. While several rigid powered exoskeletons have been developed^14,17,30^, user benefits have been demonstrated primarily for passive devices^31,32^. Most passive exoskeletons use springs to unload the arm in loaded conditions and rigid structures for stabilization to transfer forces to the shoulder, back, or pelvis. An example of a passive exoskeleton is the PAEXO (OttoBock), which assists overhead work by transferring a portion of the total load weight to the pelvis^33^. The design focus and success of these devices are understandable as passive exoskeletons require no actuators, electronics, or batteries. These devices can therefore be lightweight and used autonomously for long periods with little safety risk to the user and few sensitive parts that may fail or require maintenance.

Based on recent reviews^31,32^, passive upper limb exoskeletons have been found to reduce muscular effort during occupational tasks, including quasi-static (overhead drilling and assembly) and dynamic tasks that involved the elevation of the arms (lifting, stacking). However, antagonistic muscular effort can increase^31^, though there is limited and insufficient evidence regarding the impacts of these exoskeletons on kinematics and fatigue^32^. While passive systems have shown benefits, reviews have found insufficient evidence to support the use of active exoskeletons. Included active devices were an exoskeleton (9.2 kg) that uses McKibben muscles to assist the elbow, shoulder, and trunk^34,35^, and the Lucy exoskeleton that uses actuated pneumatic cylinders to support the shoulder^36^. A semi-passive shoulder exoskeleton (12 kg) was also included, which uses a motor to change the assistance force of two springs^37^. Recently, another semi-passive concept demonstrated reductions in user’s shoulder muscle activity and heart rate during overhead work (H-PULSE, 5 kg^38^).

In addition to these exoskeletons, semi-passive clutch based^39,40^ and numerous motor-powered systems have been developed in recent years^41^. Active exoskeletons are in general heavier than their passive counterparts that weigh 1.8–6.5 kg^33^. For both passive and active devices alike, the overall human effort for moving or carrying the exoskeleton increases with device weight, regardless of localized effort reduction. Similarly to the lower limb, lightweight upper limb exoskeleton designs with minimal inertia are required and exosuits have therefore been investigated for the upper limb as well^42–46^. For example, an exosuit to assist the elbow (2 kg to assist one elbow^43^) reduced the biceps brachii activity (65%) and the biological elbow moment (60%) when holding 2.3 kg with a maximum assistance torque of 6 Nm^47^. Further, a reduced onset of fatigue was found when isometrically holding a weight of 1 kg.

**Figure 1:**
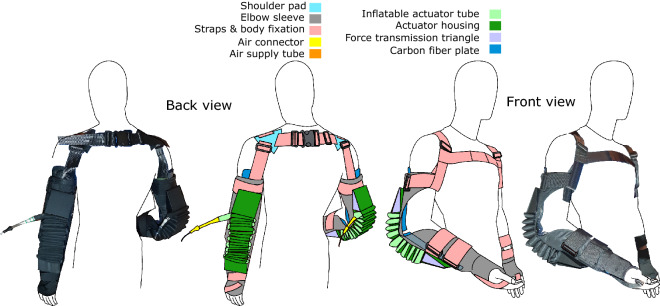
Pneumatic soft elbow exoskeleton (Carry) front and back view.

Pneumatic actuation has also been used in upper limb exoskeletons^28,41^. Aside from McKibben-like pneumatic muscles where linear shortening is used to assist the rotation of the desired joint, soft textile-based pneumatic actuators have been introduced^28,48^, which use the deformation of the actuator itself to rotate the joint. With these actuators, exoskeleton designs with a soft human–machine interface and soft actuation are possible. A soft interface and actuation has been used for exoskeletons assisting the shoulder^49,50^, elbow^51–53^, wrist^54,55^, and fingers^56,57^. The soft textile-based pneumatic elbow exoskeleton of Thalman et al.^52^ demonstrated reduced biceps brachii activity (one subject) when holding 1.5 kg (43%) and 2.5 kg (63%) and when performing a dynamic range of motion test without weight (47%).

The exoskeleton state-of-the-art demonstrates that minimalist and lightweight concepts with minimal distal masses (inertia) seem most promising for providing assistance. Passive exoskeletons are able to meet these design requirements and have proven to be useful and effective in larger group studies. Active upper extremity exoskeleton concepts struggle to be lightweight, comfortable, and without movement restrictions, which limits their utility. Further, most of the systems that have proven to be useful were designed to support overhead work. Evidence of usefulness is limited for load holding and load carrying tasks, not only at the local muscle level, but also at the level of the body as a whole in terms of reduced effort and fatigue.

Here we introduce Carry, a lightweight active upper extremity exoskeleton to assist with elbow flexion during load holding and carrying scenarios. Carry combines two of the most promising concepts, a soft human–machine interface and soft pneumatic actuation (Fig. 1).

This article focuses on (1) user outcomes with isometric Carry assistance, and (2) Carry actuator performance outcomes and investigations regarding the operation dynamics and the autonomous usability.

User outcomes were evaluated with a tethered version of Carry, which was used to assist a short-duration holding scenario with different loads, a long-duration holding scenario, and a long-duration carrying scenario. For all scenarios, trials with and without the assistance of Carry were compared to investigate changes in muscle activity, metabolic cost, and fatigue. We hypothesize that Carry is able to reduce muscle activity, and consequently the metabolic cost and fatigue.

**Figure 2:**
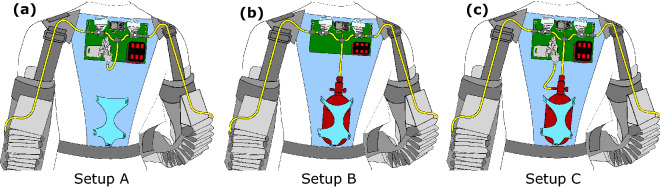
Possible future autonomous Carry setups. (**a**) Setup A is powered by a compressor, (**b**) Setup B by a pressure tank, and (**c**) Setup C by a compressor and a pressure tank.

The actuator performance analysis includes determining the relationship between Carry’s actuator elbow assistance torque and actuator pressure. Investigations on the operation dynamics and autonomous usability assume three different autonomous Carry configurations (Fig. 2). Setup A is powered by a compressor, Setup B by a pressure tank, and Setup C by a combination of a compressor and tank. Regarding the operation dynamics, the minimum possible cycle times were determined for each setup based on actuator air fill and release times and on pneumatic valve open and close times. Regarding the autonomous usability, the total amount of possible use cycles was determined for each setup with a reasonable battery size and tank volume and pressure. We also provide weight estimates for each of the autonomous setups.

## Results

### User outcomes with isometric Carry assistance

**Figure 3:**
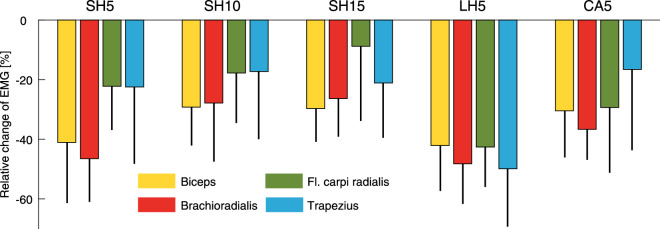
Relative change of the EMG including standard deviation from the unassisted to the assisted conditions for the short holding scenarios (SH5 5 kg, SH10 with 10 kg, SH15 with 15 kg), the long holding scenario (LH5, 5 kg), and the load carrying scenario (CA5, 5 kg).

With Carry assistance, we found relative reductions in EMG for all conditions (Fig. 3). When considering all scenarios, mean EMG was reduced by 35 ± 6% for the biceps brachii ($p=0.00059$), 37 ± 10% for the brachioradialis ($p=0.0276$), 24% ± 13% for the flexor carpi radialis ($p=0.0015$), and 25 ± 14% for the trapezius pars transversa ($p=0.0013$). The largest reductions (50%) were found for the trapezius in the long holding (LH5) scenario. With increased loads during the holding scenarios, the relative reductions in EMG partially decreased. In contrast, longer-standing times seem to increase the EMG reductions for the flexor carpi radialis and the trapezius pars transversa. When comparing the relative reductions in EMG in the long holding and the carrying scenarios, we found that carrying benefits less from Carry assistance.

**Figure 4:**
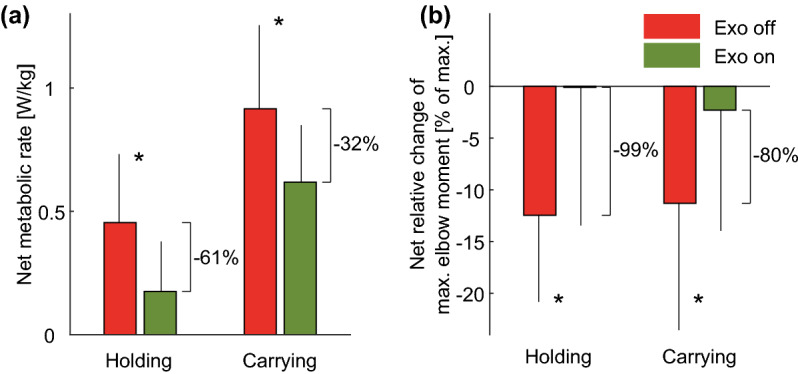
(**a**) Net metabolic rate and (**b**) relative change in the maximum elbow moment including standard deviation of the assisted (green) and unassisted (red) long holding and carrying scenarios (* $p<0.05$).

The net metabolic rate decreased from 0.45 ± 0.28 W/kg to 0.18 ± 0.2 W/kg in the long holding scenario ($p=0.0278$) and from 0.92 ± 0.34 W/kg to 0.62 ± 0.23 W/kg in the carrying ($p=0.0371$) scenario, which is a relative reduction of 61% and 32%, respectively (Fig. 4a). For reference, standing and walking with the same arm position but without the 5 kg load required 1.62 ± 0.28 W/kg and 4.35 ± 0.46 W/kg, respectively.

The mean maximum elbow flexion moment, for all MVIC pre- and post-tests of all subjects, was found to be 80 ± 24 Nm. The MVIC decreased from pre- to post-test due to fatigue in the long holding scenario by 12 ± 8% and 0 ± 13% for the unassisted and assisted condition, respectively (Fig. 4b, $p=0.0073$). For load carrying, the MVIC decreased from pre- to post-test by 11 ± 12% in the unassisted condition and by 2 ± 12% in the assisted condition ($p=0.0269$). Thus, the assisted condition showed no fatigue (99% less) in the long holding scenario and 80% less fatigue in the carrying scenario.

### Actuator performance, operation dynamics, and autonomous usability

**Figure 5:**
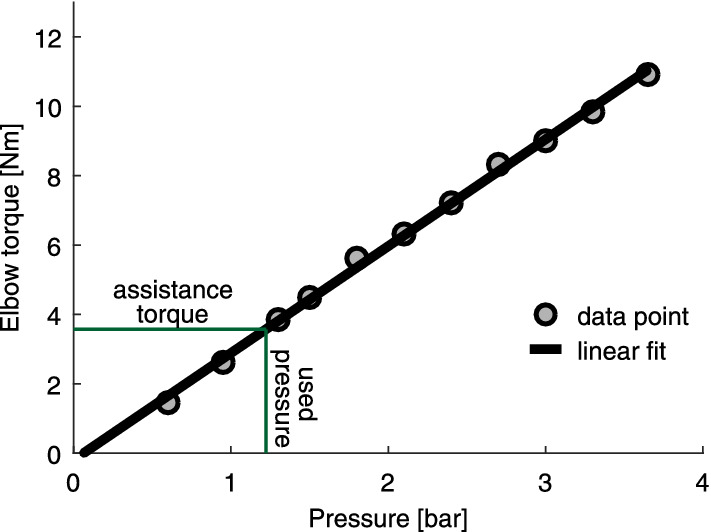
Relationship between actuator pressure and assistance torque. At 1.22 bar, one Carry actuator provides 3.6 Nm of elbow assistance torque.

A linear relationship of the elbow flexion assistance torque and the soft actuator air pressure was found ($y=3.08x-0.2$, R$^2$ = 0.998, Fig. 5). The highest achieved pressure was 3.65 bar, which corresponds to 11 Nm of elbow flexion torque. The grand mean of the pressure used within all experimental trials was 1.22 ± 0.03 bar (1.3 bar desired), which corresponds to an elbow flexion torque of 3.6 Nm per arm.

Filling a single Carry-mounted actuator with 1.3 bar using the mobile compressor required 40.4% of the time of a single unmounted actuator (3.72 L at 1.3 bar). Based on the given Carry-unmounted actuator volume, for two mounted actuators, the required air volume $V_{Carry}$ was estimated to be 3 L (at 1.3 bar).

**Figure 6:**
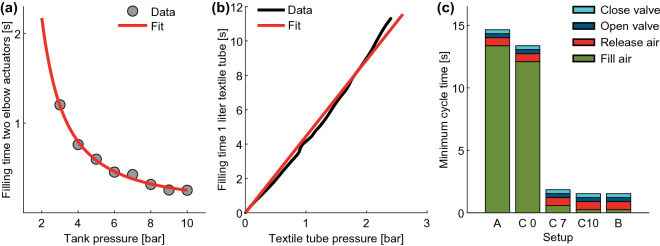
Exoskeleton operation dynamics including (**a**) the tank pressure to Carry filling time relationship, (**b**) the textile tube pressure (one liter) to mobile compressor filling time relationship, and (**c**) the Carry cycle times with respect to the supply mode of Setup A (compressor), C0 (tank almost empty), C7 (tank refilled to 7 bar), C10, and B (both tank above 10 bar).

When using the mobile compressor to fill a one-liter actuator to 2.6 bar, a linear relationship between fill time $T_{comp}$ and the injected air volume (*V* in L) was identified ($T_{comp} = 4.446 \cdot V$, R$^2$ = 0.991, Fig. 6b), which corresponds to a fill rate of 0.225 L/s. To fill $V_{Carry}$, a $T_{comp}$ of 13.37 s is required.

When using a pressure tank with up to 10 bar, the fill time of $V_{Carry}$ can be determined by $T_{tank} = 6.131\cdot x^{-1.55}+0.07846$ (R$^2$ = 0.994). $T_{tank}$ was found to increase with reduced tank pressure and the shortest $T_{tank}$ (0.25 s) was found at 10 bar (Fig. 6a). The refilled tank of Setup C drops from 7 bar to 3.2 bar when filling $V_{Carry}$ once. Based on the resulting average pressure of 5.1 bar, $T_{tank}$ was estimated to be 0.57 s. In comparison, at the desired actuator pressure of 1.3 bar, releasing $V_{Carry}$ from both elbow actuators required 0.65 s ($T_{release}$). Next to the times that are required for the air flow, the times to open ($T_{openvalve}$) and close ($T_{closevalve}$) the pneumatic valves were identified to be 0.16 s and 0.31 s, respectively.

Based on the autonomous setup specific time constraints, the minimum possible cycle times for each setup were determined. With the use of a mobile compressor, the minimum $Cycletime_{A}$ was determined to be 14.6 s. For tank pressures above 10 bar, the minimum $Cycletime_{B}$ and $Cycletime_{C10}$ were determined to be 1.5 s (Fig. 6c). After refilling the tank to 7 bar, the minimum $Cycletime_{C7}$ was identified to be 1.8 s, and this increases to a $Cycletime_{C0}$ of 13.4 s in the worst case when the tank is almost empty (already filled 0.32 L within $T_{release}$ and $T_{openvalve}$).

With a battery capacity of 40 Wh and a stored tank air volume of 222 L, the total amount of use cycles was determined. With the battery-powered mobile compressor, $Cycles_{A}$ was measured to be 119, with the tank $Cycles_{B}$ was estimated to be 73, and for the combination, $Cycles_{C}$ was estimated to be 192.

## Discussion

### Exoskeleton performance

A pneumatic elbow exoskeleton (Carry) with a soft human–machine interface and soft actuation was designed and used to assist users in isometric load holding and load carrying scenarios. Based on bench testing, the used actuator pressure of 1.22 bar corresponds to an elbow assistance torque of 3.6 Nm for each arm. For reference, for a mean forearm length of 0.34 m, holding loads of 5, 10, and 15 kg requires 16.7 Nm, 33.4 Nm and 50 Nm, respectively. When providing 7.2 Nm of assistance with both Carry arms, the assistance provided is 43%, 22%, and 14%, respectively. The assistance torque led to significant reductions in muscle activity and metabolic rate. The reduction of the biceps activity is in line with a similar, previously investigated concept evaluated on a single subject^52^. In addition, reductions of the muscle activity were not only found for the muscles for elbow flexion, but also for a muscle that stabilizes the shoulder (trapezius) and a muscle that stabilizes the wrist (flexor carpi radialis). Surprisingly, while the combined assistance of both Carry arms contributed 7.2 Nm of the required 16.7 Nm to hold the 5 kg load, almost no fatigue was found in the assisted long holding scenario, and fatigue was reduced by 80% in the assisted load carrying scenario. The EMG reductions for the three different load conditions of the short holding scenarios demonstrate that Carry is also able to assist for a wide range of weights (5 kg to 15 kg). The results partially indicate that less user benefit can be expected for increased loads given the same assistance torque. From the short- versus long-hold scenarios, we find that the benefits of Carry increased with hold duration. This may be due to users having a greater reliance on assistance for longer duration holds, and there may be a training effect such that users may be able to better adjust to the provided assistance over time. The EMG data indicates that such an effect could be pronounced for supportive stabilizing muscles. While the biceps and the brachioradialis, which are distinct elbow flexors, did not show reduced benefits over time, the flexor carpi radialis (stabilizes wrist next to elbow flexion) and the trapezius (stabilizes shoulder) show reductions. Stability could be also a reason for the reduction in benefits of the carrying condition CA5, compared to the long holding condition LH5. Both conditions require the muscles to support the weight and to stabilize the joints. We believe that the stabilization role is larger within the carrying condition as the human center of mass shifts horizontally and vertically during walking. Carry might support the stabilization function less than the weight support function, which could lead to observed reduction in benefits for the carrying condition. Another possible source for the difference in the relative change of EMG between LH5 and CA5 could be a slightly changed posture of the trunk and the upper limbs.

Based on NIOSH recommendations, workers should not exceed 2.2 kcal/min for lifting tasks performed for 2–8 h^11^. For comparison, our normalized metabolic rate (W/kg) was denormalized and multiplied by a factor of 0.01434 (1 W = 0.01434 kcal/min). Results show that energy expenditure was reduced during holding from 2.49 kcal/min without assistance from Carry to 2.16 kcal/min with assistance. We therefore believe that this improvement could assist in the prevention of systemic or aerobic fatigue, and possibly local muscle fatigue that may increase the risk of lifting-, holding-, or carrying-related joint degeneration and pain. Populations with a reduced maximum oxygen uptake may benefit most from reduced energetic requirements^24^. Further, with increasing age, adults might increasingly benefit from the joint torque assistance as their maximum joint torque is reduced and the loss rate of the maximum joint torque during a load holding or carrying scenario is increased^2,24^.

### Pneumatic pressure, user benefits, and comfort

The pressure used in Carry during the human experiments was 1.22 bar, which is about one third of that realized in bench testing (3.65 bar). While the assistance with two arms at 1.22 bar produces 7.2 Nm of torque and 100% support for a 2.2 kg load, 3.65 bar produces 22 Nm of torque and 100% support for a 6.6 kg load. With increasing assistance torque, we expect increased benefits for the user as was found for a semi-passive shoulder exoskeleton^38^. At a pressure of 3 bar, an elbow assistance torque of 27.6 Nm was achieved with a comparable pneumatic design^52^. Performance differences could be due to the evaluation method and due to an increased contact area between the actuator chambers, which increases the torque for similar pressures. Carry uses a single folded actuator textile tube. In contrast, Thalman et al.^52^ used an array of independent textile chambers, where each had its pneumatic connector into an air supply tube. Therefore, the number of the air supply tubes in Thalman et al. is equal to the number of the textile chambers used to build the elbow actuator. This difference leads us to benefits with respect to cost, simplicity in construction, maintenance, robustness and dimensions. While producing slightly lower torque, the single actuator tube in Carry allows for a minimalist and lightweight design. The actuator pressure is limited by user comfort as the actuator forces are applied to the arm and shoulder. With the pressure used in the human experiments, no subjects reported major discomfort during the evaluation. However, preliminary tests revealed that uncomfortably high forces were felt at the locations of the force transmission triangles. Although a sub-optimal solution, this discomfort was decreased by adding curved carbon fiber plates (shin pads) to distribute forces over larger areas. The use of pneumatic soft padding instead of carbon plates may improve force distribution, further reduce weight, and help to achieve an entire soft interface design. Such padding could be sewed to match the shape of the back of the arm when inflated. One pump could be used to fill the pneumatic actuator and the upper and forearm padding in parallel. Additionally, by covering more distal areas such as the wrist, the lever arm to the elbow can be increased, which could further reduce the local arm pressure. Future work should also investigate how a pneumatic textile-based force transmission triangle changes the performance of Carry. If increases in the actuator pressure are possible without reducing the user’s comfort, the limits of the usable actuator pressure have to be explored. During bench testing, the actuator broke at 3.9 bar as the actuator become loose from the air connector. Improved fixation could easily avoid this failure and pressures above 3.9 bar should be achievable. However, the pressure limit of an improved actuator design is currently unknown and it is unclear how this affects user comfort. In line with our experience, others reported as well that for soft human–machine interfaces, the limiting factor is expected to be the user comfort rather than the actuator torque^47^.

### Dynamic usability

The investigations of Carry were performed for isometric conditions with a constant elbow angle. Such scenarios could also be assisted with a string-based passive exosuit^58^. However, we see multiple advantages for using actuation. Exoskeleton assistance can be switched on and off, for example, by voice-based control^59^. Both hands could be used to manipulate the object of interest and no time would be wasted to manually attach the strings^58^. While a string-based exosuit would require manual adaptation to assist at other elbow angles, actuators allow for different support angles. Such a concept could also be realized by a clutch-based mechanism in combination with the string-based exosuit solution (semi-passive). However, a string-based solution^58^ would reduce the number of holding and carrying positions, and it would restrict the width of objects that are carried on the forearms. One advantage of an active exoskeleton that goes beyond the passive or semi-passive solutions is the controlled (torque adaptation) assistance in dynamic scenarios. To estimate the operation dynamics of Carry, a series of performance tests were conducted for different possible autonomous configurations. We found that at a $V_{Carry}$ of 3 L, Setup A (compressor only) would require a minimum cycle time of 14.5 s. A much shorter cycle time of 1.5 s was determined for Setup B (tank only) and C (tank and compressor) with tank pressures above 10 bar. For the refilled tank of Setup C (max. 7 bar), cycle times of 1.8 s to 13.4 s were found. A cycle time of 1.5 s seems reasonable for a variety of load handling and manipulation tasks. However, different lifting techniques (e.g., squat, stoop) were found to require about 1.3 s^26^. At this point, it is unclear what the optimal torque assistance profiles should look like for such dynamic movements, and how much users could benefit from sub-optimal assistance torques. We speculate that most movements will already benefit from sub-optimal assistance while the user provides the rest of the required total torque. To optimally assist dynamic repetitive movements, which likely include a full range of motion of the arm, flow rates that allow for releasing and filling the air in less than the desired cycle time are required. Our tests revealed that with tank pressures above 10 bar, 0.25 s is required to inject the air. However, 0.65 s is required to release the air, and opening and closing the valves requires 0.63 s in total. The valves used in our design were made to perform the static holding and carrying tasks and we believe that reductions in the time to change the valve states should be possible with other or improved valves. It seems most difficult to reduce the time to release the air. It has to be investigated how active or passive elbow extension will reduce the release time and if users can take advantage of the resistance during the release of the air (e.g., when lowering loads). Preliminary tests revealed that larger inner tube, connector, and valve diameters and a faster valve motor can be used to improve the release and fill times. For example, increasing the inner diameters from 4 mm to 6 mm reduces the release time by 26%, and using no valve reduced the release time by 23%. Tank pressures above 10 bar could be used to further reduce the fill time, as our experiments revealed a reduced fill time and no saturation with increases in tank pressure (Fig. 6a). A reduction in the actuator volume could also help to improve the Carry operation dynamics. To avoid losses in the assistance torque, increasing the contact area between the chambers by increasing the number of chambers could be explored.

### Autonomous design weight estimations

As the observed reductions in muscle activity, metabolic cost, and user fatigue are highly promising, an autonomous design of Carry is of interest. Such a design will increase the exoskeleton mass, which might eliminate the user benefits seen here. The current mass of Carry with two arms is 1.85 kg. We can see reducing this mass by using inflatable padding instead of carbon plates or by reducing the carbon plate mass (e.g., cutting unnecessary areas, thinner plate) by at least 0.3 kg. Further, the ends of the actuator tubes could be placed in a triangle-like, textile-based actuator housing to reduce the force transmission triangle mass from 0.35 kg to about 0.05 kg. A mass reduction of about 25% is possible for the shoulder straps as well. Future device generations are therefore estimated to have a total mass of 1.2 kg for the user-machine interface and the actuators. With the help of the Setups A, B, and C that were used to investigate the operation dynamics, mass estimations for an autonomous design can also be made. For Setup A (compressor only), an additional 1.52 kg would be required, which consists of a compressor (0.59 kg), compressor battery (0.43 kg), two motorized three-way valves (0.135 kg), a pressure sensor and electronics (0.08 kg), electronics battery (0.1 kg), and air tubes and connectors (0.05 kg). For Setup B (tank only), the compressor and the compressor battery of Setup A can be replaced by the tank (0.77 kg) and the necessary air mass (0.29 kg for 222 L at 1.3 g/L). Setup C (compressor and tank) requires the filled tank be added to Setup A. Thus, Setups A, B, and C would require total masses of 2.7 kg, and 2.8 kg, and 3.8 kg, respectively. The required additional components could be placed at the back (Fig. 2). Literature shows that an additional mass of 1 kg at the trunk will increase the metabolic rate of regular walking between 1.1 and 2%^60,61^, which is about 0.04–0.06 W/kg of the 3.2 W/kg measured for regular walking^60^. If we assume approximately 3 kg of additional mass is required for the autonomous exoskeleton, an estimated increase of 0.12–0.18 W/kg for the walking scenario will still be below the reductions of 0.27 W/kg for the holding and 0.3 W/kg for the load-carrying scenario, which were both performed at a low level of the possible actuator assistance torque. The increases are expected to be much lower for standing as standing requires about 1.35 W/kg and an additional 10 kg in a backpack increases this value by only 0.04 W/kg^62^. Considering all design thoughts together, each autonomous setup should provide local (reduced muscle fatigue) and global (reduced metabolic expenditure) assistance. However, slightly larger benefits are expected for use cases where a tethered system could be used (i.e., fixed workplace), as used in our experiments. If a system mass of 3 kg for an autonomous Carry can be achieved, this will be close to passive contenders such as PAEXO (1.8 kg^33^), and this would be in the range or slightly below other promising designs, such as the motor-powered exosuits (4 kg for two elbows^43^) or semi-passive systems (5 kg H-Pulse^38^). Use cycles of greater than 73 seem reasonable for mobile applications, and this could be doubled with a 0.4 kg increase in either the battery or tank weight.

### Methodological considerations

The evaluation of the exoskeleton was performed without comparing it to a condition without wearing the exoskeleton. This decision was made to limit fitting-related losses in data quality (e.g., position of human–machine interface, EMG sensor replacements) for the number of subjects evaluated, and because we did not expect larger differences for the lightweight tethered design in the static assistance tasks. As this study showed substantial user benefits, dynamic evaluations for a future autonomous version with increased mass should include such a comparison.

Next to being able to activate and deactivate the exoskeleton assistance in a certain time frame, many scenarios in which assistance could be useful require elbow joint range of motion, though this was not evaluated in this work. Similar elbow exoskeleton concepts found range of motion restrictions of 7–46%^51,52^.

### Summary and conclusion

We evaluated the performance of the elbow exoskeleton Carry, which uses soft pneumatic actuation and a soft interface to assist users during load holding and carrying. In this preliminary work with Carry, a moderate air pressure of 1.2 bar was applied, which was able to assist users’ elbow flexion torque by 43% when holding or carrying 5 kg. With this assistance, muscle activity, metabolic rate, and fatigue were significantly reduced.

A bench test revealed that the actuator pressure used was a third of the maximum possible pressure, which can be easily increased further. With a greater pressure, we expect increased physiological benefits. However, the limiting factor for the increase in pressure is user comfort, which is mainly influenced by the force distribution between the actuator and the arm. To increase the actuator pressure and maintain user comfort, we propose to use large actuator-like pneumatic force distribution plates placed distally on the forearm to increase the leverage and to reduced the local applied force.

Testing of the operation dynamics revealed that the current design has a reasonable actuator fill time at tank pressures above 10 bar, but the release time and the times to open and close the valves could limit dynamic use. However, it is possible that even sub-optimal assistance may result in substantial user benefits. The experimental results indicate that a variety of holding, carrying, and manipulation tasks with lower activation-deactivation frequencies could benefit from the proposed Carry designs. Improved valve designs, increased limits of the pressure regulator, increase connection diameters, increased tube and valve inner diameters, and an improved chamber contact area to actuator volume ratio could be explored to improve the operation dynamics.

The analyses for the autonomous design revealed that, similar to lower limb exosuits, soft interfaces allow for a lightweight upper limb exoskeleton design. Based on the use case, system weights of 1.2 kg for a tethered and 2.7 kg to 3.8 kg for an autonomous Carry should be expected.

## Methods

### Elbow exoskeleton

The soft elbow exoskeleton Carry (Fig. 1) has a weight of 1.85 kg (Table 1) and includes one tethered soft pneumatic actuator for each elbow^48^. Each actuator is made with a TPU bladder (Thermoplastic polyurethane) encased by a textile (Twill-Polyester, 90/180 den, impregnated, 180 g/sqm) tube. Upon pressurization, the inflated tube has a circular cross-section diameter of 0.045 m, a tube length of 1.8 m, and an inflated volume of 2.86 L.

The actuator tube is folded and fixed by an actuator housing of the same textile. The actuator housing also covers force transmission triangles, which are used to redirect forces from the actuator (towards proximal and distal ends of the limb) to provide an elbow flexion torque.

The human–machine interface is used to fix the actuator on the human elbow. The main structure of the interface is the elbow sleeve, which includes two carbon plates, one at the back of the upper arm and one at the forearm, to improve user comfort by distributing the actuator forces over the surface of the arm. The inner surface of the sleeve (skin contact) is coated with silicon to increase friction. The friction reduces slipping of the sleeve towards the elbow and rotations of the sleeve around the arm axis in order to ensure the actuator will act in line with the elbow axis.

Further, textile straps (Security-webbing, Polyester, braid width 50 mm, breaking strength 2200 daN, thickness 1 mm, 56 g/m) with plastic buckles are used to minimize slipping effects and to adapt Carry to the user’s size and shape. Two straps are used at the forearm to reduce sliding of the forearm sleeve towards the elbow. This is done by increasing the surface pressure for the silicon coating and by limiting movement from the cone-shaped forearm. Sliding towards the elbow, especially when Carry is not active, is also minimized by a strap that crosses the hand. The silicon coating, the forearm straps, and the hand strap are the distal anchor points. The upper arm strap is mainly used to increase the surface pressure for the silicon coating to avoid sleeve rotation around the upper arm axis. The shoulder pad (Twill-Polyester, 90/180 den, impregnated, 180 g/sqm) is used to prevent slipping of the upper arm sleeve towards the elbow. This is connected by straps in the front and the rear of the arm and is used as the proximal anchor point. Straps in the front and the rear of the user’s torso limit lateral slipping of the shoulder pads.

**Table 1:** System components worn by the user.

Component	Quantity	Mass (g)
Inflatable actuator tube	2	100
Elbow sleeve and fixation, actuator housing	2	238
Force transmission triangle	4	87
Carbon plates (pair) with cushioning	2	288
Shoulder straps	2	124
Total mass		1848

### Actuation system and sensors

A stationary compressor with a 5 L tank (Airliner 5 Go, Aerotec, max. pressure 10 bar) was used to provide the required pneumatic air pressure for the human experiments and for most bench tests. The compressor is connected to a T-connector (IQST 80, 8 mm), which splits the air supply for each actuator. On each side of this T-connector, a customized inlet valve (IQSKH 80, 8 mm), another T-connector, and a customized outlet valve (IQSKH 80, 8 mm) are connected in series. The second T-connector links to the air connector of the actuator tube. All valves and connectors are connected with 0.07 m long air supply tubes (Polyurethane, inner/outer diameter: 6 mm/8 mm). The air supply tube length was changed to 2 m for the user experiments to allow transitions between the different evaluation setups. The customized valves are equipped with a servo motor (HS-475HB, Hitec) to control the air flow (100 Hz) by a microcontroller (ESP32 NodeMCU, Espressif Systems). The valves are fully opened at 0° and fully closed at 72°. The microcontroller is also connected to the analog output of two pressure sensors (40PC150G1A, Honeywell) to measure each actuator’s pressure (100 Hz) at the end of the actuator tube.

### Exoskeleton control

Subjects were instructed to position their arms at the target position before each experimental trial with Carry. Following, the soft actuators were pressurized manually with a switch (operated by one of the authors), which triggered the inlet valves to open until a desired soft actuator air pressure of 1.3 bar was achieved. Afterwards, the experimental trials with Carry assistance were performed. At the end of each experimental trial, the soft actuators were depressurized manually with a switch, which triggered the outlet valve to open and release the pressurized air.

### Actuator torque–pressure relationship

**Figure 7:**
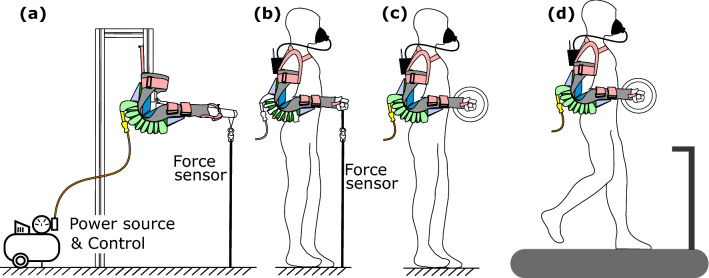
Carry exoskeleton experimental setups for investigating: (**a**) the relations between elbow flexion torque and pneumatic actuator air pressure during bench testing, (**b**) MVIC as a measure for fatigue, (**c**) changes in muscle activity in the short holding scenario (5 kg, 10 kg and 15 kg), and changes in muscle activity and metabolic cost in the long holding scenario, and (**d**) changes in muscle activity and metabolic cost in the carrying scenario. The power source and electronics were placed offboard in all setups.

A bench test (Fig. 7a) was performed to determine the relationship between the actuator pressure and elbow assistance torque. Aluminum profiles were used to represent the upper and forearms, the human elbow was represented by a hinge joint, and hard rubber padding was used to match the diameter of the aluminum profiles to that of one of the subjects. At the front of the forearm profile, a rope that was instrumented with a force sensor (ZNLBM-1, Bengbu Zhongnuo Sensor, China) was connected to the ground. The length of the rope was set to achieve angles of 90° at both the elbow and the forearm-rope junction when pressure was applied. During the test, the pressure was increased from a starting pressure of 0.6 bar in steps of 0.3 bar (10 s at each increment) until the actuator failed. The forces were measured at 20 Hz and converted to elbow torque using the lever arm (0.48 m). Torques to hold the weight of the artificial forearm were determined (in part by weight and in part by center of mass lever arm) and added to the elbow torque. Mean elbow torques and pressures of each pressure increment were calculated following experimentation and were used to determine a single degree polynomial fit.

### Exoskeleton operation dynamics and autonomous usability

The dynamic and autonomous capabilities of Carry depend highly on the pressure source and the achievable air flow. Within this work, we theoretically analyzed the operation dynamics and autonomous capabilities of three different Carry configurations (Setups A, B, and C, Fig. 2) based on measured performance data. For each configuration, the air fill time, the release time, and the times to open and close the valves are required to determine the overall minimum possible cycle time. In addition, the possible total number of cycles was determined, where this is limited by the air source. Air source limitations of a mobile system could be due to the battery or air tank capacity.

In Setup A, a mobile compressor is directly connected to both Carry actuators. In Setup B, only a tank is connected to both Carry actuators. In Setup C, a mobile compressor, a mobile tank, and both Carry actuators are connected in series and it is assumed that the compressor can charge the tank continuously while the actuators are releasing air.

For the performance estimations of all setups, the air volume $V_{Carry}$ (in L) of both mounted actuators, at a pressure of 1.3 bar, is required. The inflated volume of a single actuator tube in an unmounted condition was calculated to be 2.86 L (at 1 bar). $V_{Carry}$ was determined by measuring the filling times to 1.3 bar, once each in the unmounted and mounted (elbow angle of 90°) conditions. Assuming a linear relationship between air volume and filling time, $V_{Carry}$ can be estimated.

To determine the compressor-based actuator fill time $T_{comp}$ (in s), a commercial battery-powered autonomous compressor was selected (PAK 20-Li B2, Parkside, compressor 0.59 kg, battery 0.43 kg, max. pressure 7 bar). The autonomous compressor was used to fill a one-liter actuator with pressure starting at 0.2 bar and ending at 2.6 bar, while the fill time was measured. The recorded pressure was filtered (zero-lag, second-order Butterworth, cut-off 1 Hz). The relationship between time and the volume of injected air was then determined by a linear fit.

To determine the actuator air release time $T_{release}$ (in s), Carry was fit to a test bench (elbow angle 90°). After filling the actuator with 1.3 bar, the air was released while the pressure was recorded. The recorded pressure was filtered (zero-lag, second-order Butterworth, cut-off 10 Hz), and the time and the pressure change in between passing an upper (0.01 bar less than mean of starting level) and lower (0.3 bar above the mean of final level) threshold were determined. With the identified mean rate (pressure change per time), $T_{release}$ was scaled to a pressure change of exactly 1.3 bar. The mean of eight trials is reported.

The tank pressure-based actuator fill time $T_{tank}$ (in s) depends highly on the current tank pressure. To determine $T_{tank}$, both Carry actuators (elbow angle 90°) were filled using tank pressures in between 3 and 10 bar (1 bar steps, stationary compressor). The valves were opened (and closed) for a fixed amount of time to achieve an actuator pressure of about 1.3 bar. The pressure was recorded and post-processed similar to $T_{release}$ with the difference of a lower threshold of 0.1 bar. A two-term power series model (Matlab) was used to fit the tank pressure to $T_{tank}$ relationship. For tank pressures above 10 bar, $T_{tank}$ is assumed to be constant at 10 bar to match the limited output of a pressure regulator.

The experimental data that was used to determine $T_{release}$ and $T_{tank}$ could also be used to determine the time required to open the valve ($T_{openvalve}$) as in parallel to the pressure, the motor command was recorded. $T_{openvalve}$ was defined as the time difference between the motor command to open the value and when air began to flow. The mean $T_{openvalve}$ of the 16 trials from the injection and release of the air is reported.

To determine the time to close a fully open valve ($T_{closevalve}$), the data from the $T_{tank}$ investigations was used. For the pressures of 4, 5, and 6 bar, the valve was completely open during the filling process (observed by video). $T_{closevalve}$ was defined as the time difference between the motor command to close the valve and when air flow ceased. The mean of the three trials is reported.

The minimum possible cycle time for Setup A ($Cycletime_{A}$) is the sum of $T_{comp}$, $T_{release}$, twice $T_{openvalve}$, and $T_{closevalve}$.

1 $$\begin{aligned} Cycletime_{A} = T_{comp} + T_{release} + 2 \cdot T_{openvalve} + T_{closevalve} \end{aligned}$$

To calculate the cycle time for Setup B ($Cycletime_{B}$), $T_{comp}$ is replaced by $T_{tank}$ at 10 bar. While the tank pressure is above 10 bar the cycle time for Setup C $Cycletime_{C10}$ is similar to $Cycletime_{B}$. If Setup C is used at the maximum usage rate, $Cycletime_{C0}$ is almost equal to $Cycletime_{A}$ with the difference that the time to open and close the valves and to release the air from the actuator can be used for charging the tank. As a result, $Cycletime_{C0}$ matches $T_{comp}$. To quantify the cycle time for Setup C after it is refilled to 7 bar $Cycletime_{C7}$, the loss in the pressure for one filling was determined based on the tank volume and $V_{Carry}$. The mean of this resulting pressure and the 7 bar was calculated and the related fill time was extracted from the $T_{tank}$ fit. This extracted $T_{tank}$ replaces $T_{comp}$ in Eq. (1) to estimate $Cycletime_{C7}$.

To determine the total number of cycles for Setup A ($Cycles_{A}$), the number of cycles was measured with the fully charged battery from the mobile compressor (20 V, 2 Ah, 40 Wh) by repeatedly filling both Carry actuators with 1.3 bar and releasing the air until the battery was empty.

For an estimation of the total number of cycles with a pre-charged tank (Setups B and C), a commercial 0.79 L tank was selected (Empire Mega Lite 48/4500). The tank can store air with a maximum pressure of 310 bar at a weight of 0.76 kg, including a pressure reducer. At an air compression factor of 1.1^63^, the tank can store 222 L of air. $Cycles_{B}$ was calculated by subtracting the minimum possible fill level of the tank to charge both actuators ($1.3\,bar \cdot 0.79$) from the total volume 222 L, and then dividing the result by $V_{Carry}$.

The total number of cycles for Setup C ($Cycles_{C}$) is the sum of $Cycles_{A}$ and $Cycles_{B}$.

### Experimental protocol

Twelve male subjects (age: 32.8 ± 7.8 years, height: 1.8 ± 0.08 m, mass: 83.8 ± 12.2 kg, forearm length: 0.34 ± 0.02 m) free of carrying- or holding task-related impairments (self reported) participated in the experiment. The institutional review board of the TU Chemnitz approved the study protocol. All subjects gave written informed consent in accordance with the Declaration of Helsinki after the nature and possible consequences of the studies were explained.

The experimental evaluation of the first Carry design consisted of two main parts. Carry was worn for all scenarios and conditions of both parts.

The aim of the first part was to evaluate weight-based changes in muscle activity with and without exoskeleton assistance. Within the first part, the short holding (SH) scenario (Fig. 7c), subjects held a dumbbell with two hands (via handles on the sides of the dumbbell) weighing 5 kg (SH5), 10 kg (SH10) and 15 kg (SH15) for a short duration of 30 s. Subjects had a break of 80 s in between each trial. After holding each with and without assistance, the weight of the dumbbell was changed based on a balanced and randomized study design. During the measurement, subjects were instructed to align the upper arm with the trunk and keep the elbow at 90°. At this angle, humans can exert their maximum elbow flexion torque^64^.

The aim of the second part was to evaluate differences in muscle activity, metabolic rate, and fatigue for holding and carrying with and without exoskeleton assistance. The second part included a long hold (LH) with 5 kg (LH5, Fig. 7c) and a long carrying (CA) with 5 kg (CA5, Fig. 7d) scenario, each with three conditions: 1) without weight and without assistance, 2) with weight and without assistance, and 3) with weight and with assistance. Experimental order was balanced and randomized, and each condition was run for 7 min. One minute before and 1 min after each condition a maximum elbow flexion moment test was performed with an instrumented rope (Fig. 7b). After the test, subjects had a break of 10 min until the next condition. The posture during the long holding and the carrying scenarios was comparable to the short holding scenario with one difference being that the weights were allowed to slightly contact the trunk. Due to the face mask, the visual assessment of the weight position was limited and the tactile feedback helped the subjects to keep the same position. Further, humans typically hold or carry objects as close as possible to their body.

### Muscle activity

The muscle activity (electromyography, EMG) of four muscles on each side of the body that were expected to contribute to performing the scenarios of interest were analyzed (2148.1 Hz) with wireless surface electrodes (Delsys, Trigno Avanti) placed based on SENIAM (seniam.org). The analyzed muscles included the short head of the biceps brachii, the brachioradialis, the flexor carpi radialis, and the trapezius pars transversa on each side of the body. The biceps brachii, the brachioradialis and the flexor carpi radialis span the elbow and contribute to the elbow flexion and/or rotation. The flexor carpi radialis is also known to flex and abduct the hand and thus controls and stabilizes the wrist as well. The trapezius pars transversa stabilizes the shoulder (by scapula). Thus, potential muscular effects for the elbow flexion, potential effects on the wrist and potential indirect effects on the shoulder are included in our analysis. The EMG processing consisted of first removing the EMG offset by subtracting each value by the mean of the whole trial. Next, the EMG data was band-pass filtered (zero-lag, fourth-order Butterworth, cut-off 20–450 Hz), rectified, and low-pass filtered (zero-lag, fourth-order Butterworth, cut-off 6 Hz). In the case of EMG artifacts (data larger than six times the standard deviation of the mean EMG of the trial), data ranging from one second before to one second after the artifact was removed (this occurred about once each 7-min trial). Within the short holding scenario, the middle 20 s of data were extracted for further analysis. For the LH5 and the CA5 scenarios, 160 s before the last 20 s of the condition were extracted. Mean EMG activity was then determined for each scenario, muscle, side, and subject. The relative changes in the mean EMG for the left and right sides of the body were calculated by Eq. (2) with the assisted and the unassisted conditions as inputs.

2 $$\begin{aligned} EMG_{rel} = -100 \cdot (1 - EMG_{assisted} / EMG_{unassisted}) \end{aligned}$$

Relative EMG changes for the left and right sides were averaged to determine a mean muscle EMG change for each subject, and grand means were then computed across all subjects for each muscle.

### Metabolic rate

Metabolic rate was determined for the LH5 and the CA5 scenarios by means of indirect calorimetry (K5, Cosmed). Oxygen consumption and carbon dioxide production were recorded at 0.1 Hz (mix chamber), and used to calculate the metabolic power using a modified Brockway equation^65^. The metabolic power was normalized to the individual subject mass. The subject-specific scenario mean was determined based on the last 3 min of the total 7 min trial for each scenario. The net metabolic rate was obtained by subtracting the metabolic rate from the same scenario (either standing or walking) without the 5 kg weight and without exoskeleton assistance. The relative changes in net metabolic rate between the assisted and unassisted conditions were calculated similar to Eq. (2). Two subjects shortly stumbled while walking on the treadmill, and 2 min of data post-stumbling were excluded in these cases. This included data from the last 3 min of the scenarios, and fewer samples were therefore available for both cases. Grand means and standard deviations were determined based on the subject means.

### Maximum elbow moment-based fatigue

Fatigue is defined as the exercise-induced reduction of the capacity to generate maximal muscle force or power output^1,2,66^. It can be determined by direct and indirect methods^66^. The authors selected a direct method, the maximum voluntary isometric contraction (MVIC) to evaluate the fatigue. Fatigue was determined for the LH5 and CA5 scenarios by a MVIC test of the elbow flexors (Fig. 7b) where subjects were pulling on an instrumented rope (1D force sensor, NLBM-1, Bengbu Zhongnuo Sensor, China). The rope length was adjusted to achieve the intended 90° elbow angle when the upper arm was aligned with the trunk. Subjects were instructed to perform three MVICs for 5 s each with 10 s rest periods between exertions where timing was guided by a visual metronome. The forces were measured at 20 Hz and used to determine the net relative change in the maximum elbow flexion moment between the MVIC 1 min before (pre) and 1 min after the session task (post). To account for signal noise, MVIC forces were filtered with a moving average filter (sliding window of 20 frames) and extracted based on a 70 N threshold.

The extracted MVICs that were shorter than four seconds were removed (8 MVICs in total). The 60 highest force values (covering 3 s at 20 Hz) from each MVIC were used to determine the mean force of each MVIC. Based on the mean across all MVICs of each subject (36 MVICs), outliers were excluded that were below a 50% or above a 150% threshold (4 MVICs total). To note, mean MVIC dropped by about 18% from the beginning to the end of the second part of the experiment. In total, 12 of 432 MVICs were removed. Following, subject-specific means of the three consecutive MVICs were determined.

Net changes in the MVIC force were determined by subtracting pre- from post-test values. The net changes were further divided by the mean across all MVICs of each subject and they were multiplied by 100 to get the net relative change in force (% of mean MVIC). These values were multiplied by the subject-specific arm length to determine the net relative change in the maximum elbow flexion moment. Finally, the grand means and standard deviations were determined.

The relative changes between the assisted and unassisted conditions for the net relative change in the maximum elbow flexion moment were calculated similar to Eq. (2).

### Statistics

For each of the four muscles, a repeated measures MANOVA was performed to test for a difference in the relative EMG between the conditions with and without exoskeleton assistance (independent variable). Each of these four tests included both body sides (e.g. left and right biceps) and all five conditions SH5, SH10, SH15, LH5, and CA5 (in total 10 dependent variables).

A Wilcoxon signed-rank test was used to test for a difference in the net metabolic rate and the net relative change in maximum force (fatigue) between the with and without assistance conditions for LH5 and CA5.

A Holm–Bonferroni step down correction was used to address the multiple comparison problem. To simplify the presentation of the results for the reader, instead of the alpha level of 0.05, the *p* values of all eight tests (2 $\times$ force, 2 $\times$ metabolics, 4 $\times$ EMG) were adjusted.

## Data Availability

The datasets generated during and/or analysed during the current study are available from the corresponding author on reasonable request.
